# Multifunctional Fe_3_O_4_ Nanoparticles Filled Polydopamine Hollow Rods for Antibacterial Biofilm Treatment

**DOI:** 10.3390/molecules28052325

**Published:** 2023-03-02

**Authors:** Huy Quang Tran, Husna Alam, Abigail Goff, Torben Daeneke, Mrinal Bhave, Aimin Yu

**Affiliations:** 1 Department of Chemistry and Biotechnology, Swinburne University of Technology, Hawthorn, VIC 3122, Australia; 2 Department of Chemical and Environmental Engineering, School of Engineering, RMIT University, Melbourne, VIC 3001, Australia

**Keywords:** polydopamine, Fe_3_O_4_ nanoparticles, drug delivery system, fosfomycin

## Abstract

This work reports the use of mesoporous silica rods as templates for the step-wise preparation of multifunctional Fe_3_O_4_ NPs filled polydopamine hollow rods (Fe_3_O_4_@PDA HR). The capacity of as-synthesized Fe_3_O_4_@PDA HR as a new drug carrier platform was assessed by its loading and the triggered release of fosfomycin under various stimulations. It was found that the release of fosfomycin was pH dependent with ~89% of fosfomycin being released in pH 5 after 24 h, which was 2-fold higher than that in pH 7. The magnetic properties of Fe_3_O_4_ NPs and the photothermal properties of PDA enabled the triggered release of fosfomycin upon the exposure to rotational magnetic field, or NIR laser irradiation. Additionally, the capability of using multifunctional Fe_3_O_4_@PDA HR to eliminate preformed bacterial biofilm was demonstrated. Upon exposure to the rotational magnetic field, the biomass of a preformed biofilm was significantly reduced by 65.3% after a 20 min treatment with Fe_3_O_4_@PDA HR. Again, due to the excellent photothermal properties of PDA, a dramatic biomass decline (72.5%) was achieved after 10 min of laser exposure. This study offers an alternative approach of using drug carrier platform as a physical mean to kill pathogenic bacteria along with its traditional use for drug delivery.

## 1. Introduction

The utilization of mesoporous silica materials for biomedical applications, especially for controlled drug delivery purposes, has been well-documented in a number of research studies [1,2,3]. The properties that make this kind of material popular include the porous structure with a high surface area, good biocompatibility, the capability of tuning particle size and forming desired structures under specific synthesis conditions. Besides their common use as drug carrier platforms, those materials are often employed as sacrificial templates for the fabrication of polymeric hollow structures with high uniformity and a well-defined shape; however, it was commonly followed by the etching of the silica core via harsh chemical treatments [4,5].

The incorporation of Fe_3_O_4_ nanoparticles (NPs) into drug carrier systems to form a magnetic guidance system has gained substantial interest due to its low toxicity and biocompatibility [6,7]. For instance, Lu et al. [8] reported their work on the synthesis of triple porous microspheres, in which porous Fe_3_O_4_ particles served as magnetic cores being engulfed by dual porous SiO_2_ shells for the controlled delivery of 5-fluorouracil. Javanbakht et al. [9] reported the use of magnetic Fe_3_O_4_ NPs as a core structure engulfed by double-shell layers of SiO_2_ and tannic acid to co-deliver doxorubicin and methotrexate for anticancer treatment. In the majority of Fe_3_O_4_ based drug delivery systems, it is common that Fe_3_O_4_ NPs served as the internal magnetic core which is engulfed by an outer layer of silica for drug binding.

**Figure 1:**
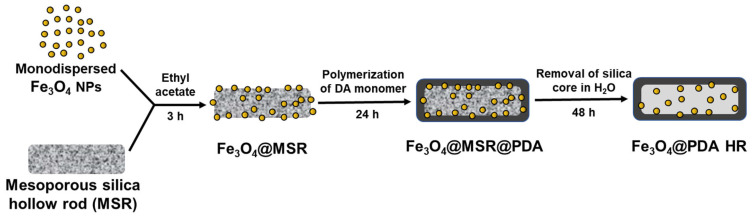
Schematic illustration for the preparation of Fe_3_O_4_ NPs filled PDA HR.

Herein, we report the preparation of a Fe_3_O_4_ filled PDA hollow rod-like structure (PDA HR) as a novel drug carrier platform. As shown in Figure 1, monodisperse Fe_3_O_4_ NPs are firstly grafted onto the surface of mesoporous silica rods, which are then coated with a thin layer of PDA. After the removal of the silica rod template, Fe_3_O_4_ filled PDA HRs (Fe_3_O_4_@PDA HR) are obtained. We chose rod-like structures as compared to spherical particles with a similar size, since rod-shaped structures have a higher drug-loading capacity [10] and cellular internalization efficiency [11,12].

The capability of using as-synthesized Fe_3_O_4_@PDA HR for drug loading and the controlled release of the payload under various stimuli, such as pH, NIR laser irradiation, and rotational magnetic field is then examined using fosfomycin as a model drug. Fosfomycin is a broad-spectrum antibiotic derived from cultures of *Streptomyces* spp. which has been used in clinical treatment to combat antibiotic resistant bacteria and their biofilm formation [13,14,15]. Additionally, the efficiency of using Fe_3_O_4_@PDA HR to deliver fosfomycin for antimicrobial purposes in vitro is further evaluated.

The utilization of magnetic activated metal nanomaterials for antimicrobial purposes has drawn attention from multiple researchers over the last few years [16]. For instance, Elbourne et al., reported the use of a magnetic field to attenuate and transform gallium-based liquid metal droplets to develop sharp-edged structures for the disruption of bacterial biofilms [17], or the utilization of magnetic nanoparticles under a magnetic field to create artificial channels for the improvement of antibiotic penetration within the biofilm structure was reported by Quan et al. [18].

Taking these into consideration, several questions have arisen regarding the movement of embedded Fe_3_O_4_ NPs under rotational magnetic fields and whether they could be used as a physical means to break down the PDA shell and induce damage to preformed biofilms, and whether the drug carrier itself could be used as a means to eliminate preformed bacterial biofilm in vitro. To answer those questions, further experiments are designed to evaluate the possibility of using Fe_3_O_4_@PDA HR to disrupt preformed bacterial biofilm under the exposure to a rotational magnetic field without using any therapeutic molecules as a proof-of-concept. Besides that, the photothermal conversion of Fe_3_O_4_@PDA HR inherited from a PDA component was also employed to evaluate its efficiency in terms of killing preformed bacterial biofilm under the exposure to NIR laser irradiation.

## 2. Results and Discussion

### 2.1. Synthesis of Monodisperse Fe_3_O_4_ NPs and Its Grafting onto MSR

**Figure 2:**
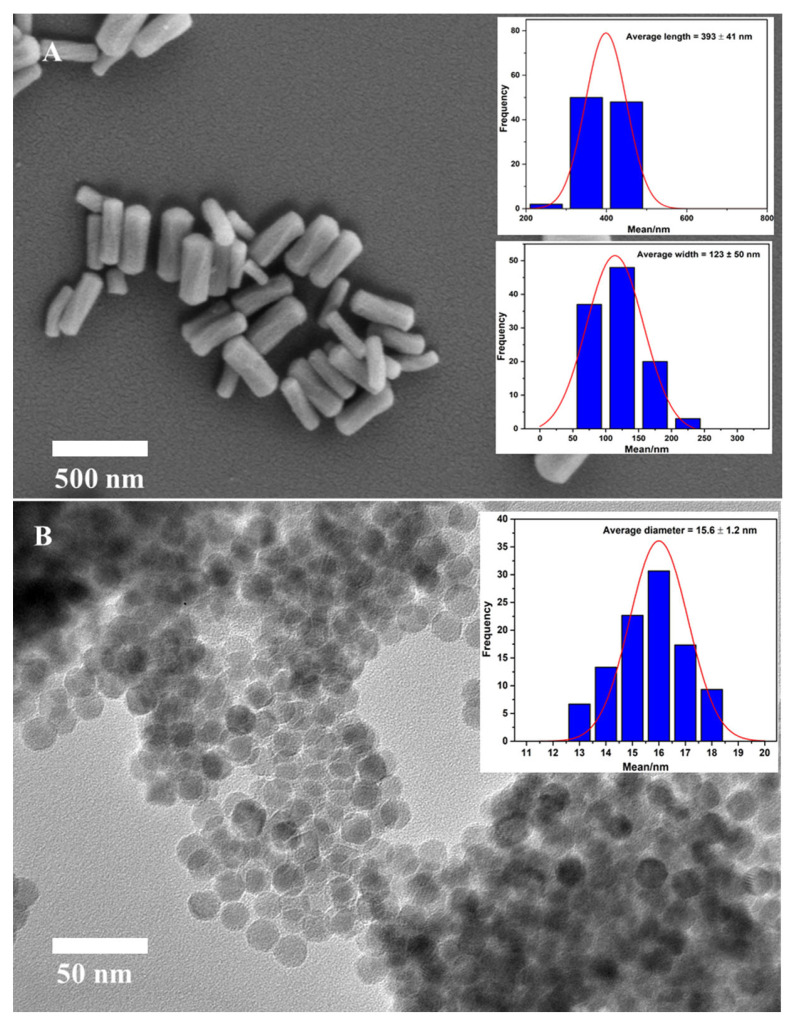
SEM image of synthesized MSR and its size distribution (**A**) and TEM image of monodisperse Fe_3_O_4_ NPs and its size distribution (**B**).

The fabrication of MSR was made use of a binary surfactant system with the aid of Pluronic F127 and CTAB, and TEOS served as the silica precursor. According to the SEM image as presented in Figure 2A, the obtained silica particles exhibit uniform rod structures with the average size of 393 nm in length and 121 nm in width. The BET analysis revealed MSR particles have a general pore diameter of 2.6 nm, and a high surface area of approximate 1600 m^2^/g.

In this work, the monodisperse Fe_3_O_4_ NPs were prepared by thermal decomposition of the iron precursor iron oleate in 1-octadecene at 320 °C. This was followed by surface functionalization with APTES to enhance its water dispersity. The morphology of monodisperse Fe_3_O_4_ was characterized using TEM (Figure 2B). As could be seen, the obtained Fe_3_O_4_ NPs have a high degree of uniformity in terms of a spherical shaped structure with an approximate diameter of 15.6 nm.

The Fe_3_O_4_ filled PDA HR was prepared through several steps including grafting Fe_3_O_4_ NPs onto carboxylic functionalized MSR via an amidation reaction, followed by the PDA coating and silica core removal. To begin with, the carboxylic functionalization of MSR was achieved through the amidation between amine functionalized MSR and the carboxyl group of succinic acid anhydride. The successful grafting of amine and subsequent carboxylic functionalized MSR was indicated by an observed zeta potential change. The zeta potential of MSR had a negative value of −33.18 mV, which was attributed by the abundance of the OH group on the silica surface. Upon amine grafting, the zeta potential shifts significantly to a positive value of 26.48 mV, which indicates the successful immobilization of the amine group on the surface of MSR. The zeta potential shifts back to a negative value of −34.25 mV after the amidation of the amine functional group and carboxylic acid group due to the ring-opening of succinic acid. This suggests that the amine functionalized MSR was successfully converted to carboxylic MSR. 

The grafting of Fe_3_O_4_-NH_2_ onto MSR-COOH was achieved via the conjugation between amine functional groups and carboxylic groups to form amide bonds. It is noteworthy that the whole conjugation process was conducted at room temperature with high efficiency, as evidenced by the very small amount of unbound MSR remaining in the solvent after magnetic separation.

**Figure 3:**
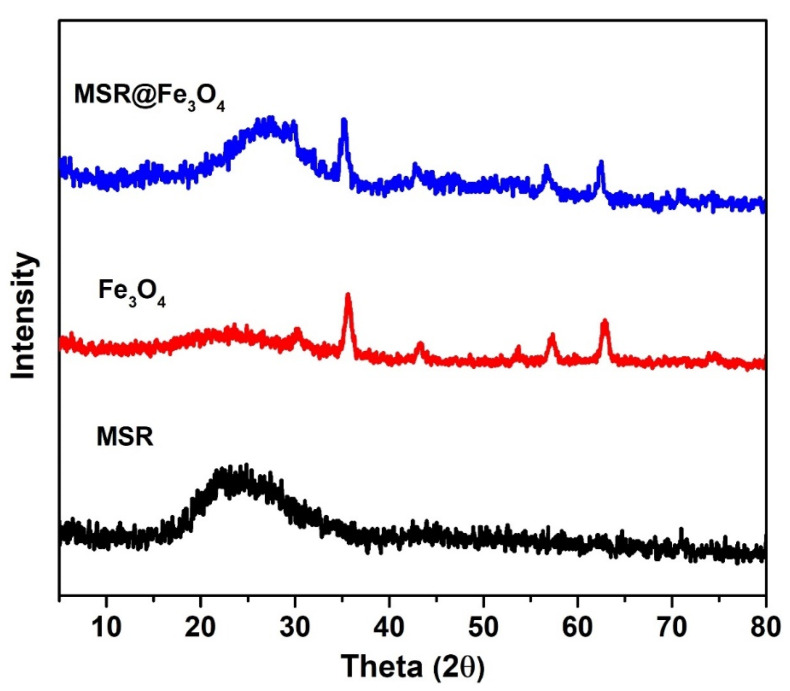
XRD pattern of monodisperse Fe_3_O_4_ NPs, MSR-COOH and Fe_3_O_4_ grafted MSR.

The successful grafting of Fe_3_O_4_ onto MSR was further confirmed by X-ray diffraction (XRD) analysis. The XRD peaks derived from monodisperse Fe_3_O_4_ NPs featured very prominently at 2ϕ values of 30.4°, 35.6°, 43.5°, 57.4°, and 63.1° (Figure 3), which are in agreement with previous studies with regards to the XRD pattern of Fe_3_O_4_ NPs [19,20]. Meanwhile, a broad peak ranging from 18° to 30° is ascribed to amorphous silica which could be observed in the samples containing silica rods [21,22]. Upon the incorporation of Fe_3_O_4_ NPs, additional peaks similar to that of bare Fe_3_O_4_-NH_2_ emerged in the XRD pattern of MSR-COOH, which implied the successful grafting of magnetite onto silica rod templates. 

**Figure 4:**
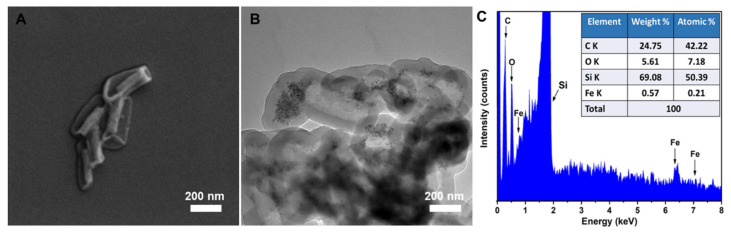
SEM (**A**), TEM (**B**) images and EDX analysis (**C**) of Fe_3_O_4_ filled PDA HR.

The Fe_3_O_4_ grafted MSR was then subjected to PDA coating and silica removal according to the PDA mediated silica dissolution process developed by our previous work [23]. The resulting structure is shown in Figure 4A. As can be seen, PDA hollow rods (HR) were obtained, which collapsed due to the loss of the silica core and drying for SEM imaging. In addition, the successful encapsulation of Fe_3_O_4_ within PDA shell was confirmed by TEM. As could be seen in Figure 4B, Fe_3_O_4_ NPs were found to remain intact within the interior of PDA HR after the removal of MSR, and this was further confirmed by EDX analysis (Figure 4C).

### 2.2. Loading and Controlled Release of Fosfomycin from Fe_3_O_4_@PDA HR

Fosfomycin was chosen as a model drug to be loaded onto Fe_3_O_4_@PDA HR. With the aid of LC-MS/MS, the amount of drug being loaded onto Fe_3_O_4_@PDA HR was calculated to be 5.7 ± 0.8 µg per mg of Fe_3_O_4_@PDA HR. Upon the successful loading of fosfomycin, the capability of triggered release of the payload from Fe_3_O_4_@PDA HR was further examined by employing a number of simulations, including pH, NIR laser irradiation and exposure to a rotational magnetic field.

**Figure 5:**
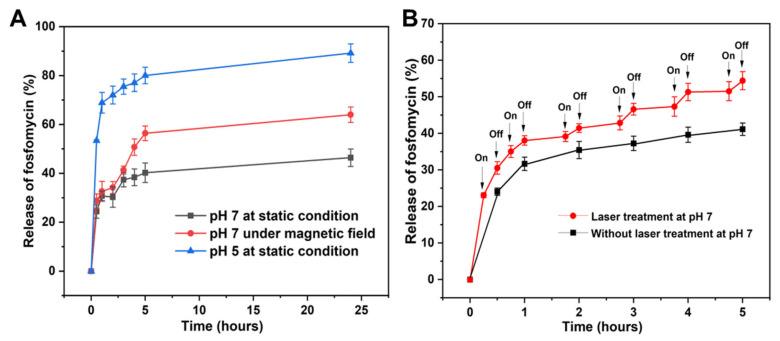
Release profile of fosfomycin from Fe_3_O_4_@PDA HR at different pH and upon the exposure to a rotational magnetic field (**A**) and upon NIR laser irradiation (**B**).

As presented in Figure 5A, the release of fosfomycin from the drug carrier was pH dependent. For both pH 5 and 7, there was an initial burst release of fosfomycin, with approximately 53.4 ± 0.3% (pH 5) and 24.4 ± 2.7% (pH 7) of fosfomycin being detected after 30 min. After that, the releasing trend of fosfomycin slowed down and gradually increased over time with nearly 89.2 ± 3.8% and 46.4 ± 3.6% of the loaded drug being released after 24 h at pH 5 and pH 7, respectively. It was noted that the amount of fosfomycin being released at pH 5 was nearly 2-fold higher than that at pH 7 at any time interval being tested. The phenomena could be rationalized by the instability of the PDA structure under acidic conditions [24,25] which might change the permeability of the PDA coating, and pH 7 was chosen as it is neutral and quite close to body pH.

Due to the incorporated magnetic Fe_3_O_4_ NPs, it was hypothesized that the movement of Fe_3_O_4_@PDA HR under rotational magnetic force might facilitate the release of loaded drugs. To test this hypothesis, the release of fosfomycin-loaded Fe_3_O_4_@PDA HR was carried out with and without exposure to a rotational magnetic field. Results indicated that the release of fosfomycin from Fe_3_O_4_@PDA HR being placed under the rotational magnetic field was higher than its counterpart. The released amount of fosfomycin under the rotational magnetic field was found to be 41.3 ± 1.6%, 56.4 ± 3.0% and 64.0 ± 3.2% after 3, 5 and 24 h, respectively, while these figures were 37.0 ± 2.9%, 40.2 ± 3.9% and 46.4 ± 3.6% without the assistance of the magnetic field (Figure 5A). We believe that the movement of Fe_3_O_4_@PDA HR upon the exposure to rotational magnetic field facilitates the detachment and release of fosfomycin from PDA HR.

In addition to the above, the ability of using NIR laser to trigger the release of fosfomycin from Fe_3_O_4_@PDA HR was examined. Under the same experimental conditions at pH 7, the release of fosfomycin under laser treatment was found to be more efficient compared to that of its counterpart. For example, after 1 h of testing, approximately 38.1 ± 1.3% of fosfomycin was detected upon laser treatment, whereas this figure in the absence of the laser treatment was just around 31.6 ± 1.8% (Figure 5B).

### 2.3. Fosfomycin Loaded Fe_3_O_4_@PDA HR for Biofilm Inhibition

In order to evaluate the efficacy of using Fe_3_O_4_@PDA HR for drug delivery purposes, the minimal inhibitory concentration (MIC) of fosfomycin and fosfomycin loaded Fe_3_O_4_@PDA HR against *S. aureus* was examined by a microbroth dilution assay. For fosfomycin, when its concentration reaches 8 µg/mL, the growth of *S. aureus* was not able to be observed after 24 h incubation. Whereas the MIC of fosfomycin loaded Fe_3_O_4_@PDA HR against *S. aureus* was determined at 1.25 mg/mL, which corresponds to the same amount of fosfomycin that was loaded onto Fe_3_O_4_@PDA HR.

**Figure 6:**
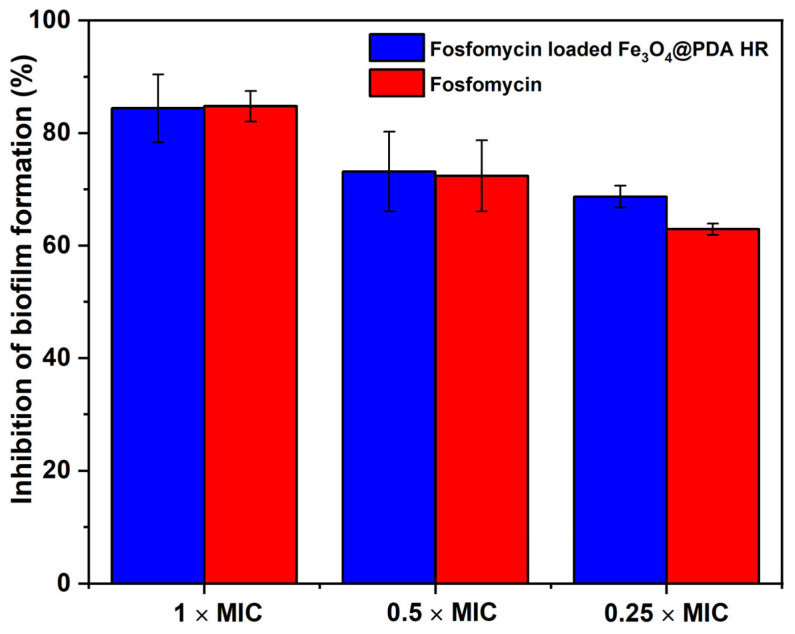
Inhibitory effect on biofilm formation of fosfomycin loaded Fe_3_O_4_@PDA HR and free fosfomycin drug molecules.

Further experiments were then conducted to evaluate the inhibition effect of fosfomycin and fosfomycin-loaded Fe_3_O_4_@PDA HR on biofilm formation. As to the results, fosfomycin exhibits an excellent antibiofilm effect regarding to *S. aureus* biofilm formation. Specifically, at the final concentration 0.25 × and 0.5 × MIC, nearly 62.9 ± 1.0% and 72.4 ± 6.3% of biofilm formation was inhibited, respectively. The inhibition rate further increased to ~85% at 1 × MIC (Figure 6). Similar results were observed in terms of fosfomycin-loaded Fe_3_O_4_@PDA HR with 68.7 ± 2.0%, 73.1 ± 7.1%, and 84.4 ± 6.0% at the final concentration of 0.25 ×, 0.5 ×, and 1 × MIC, respectively. The obtained results highlight that the fosfomycin-loaded Fe_3_O_4_@PDA HR are effective to inhibit biofilm formation, with an efficacy similar to that of free drug molecules, indicating fosfomycin retained its bio functionality after being loaded on PDA HR.

### 2.4. Fe_3_O_4_@PDA HR as Physico-Mechanical Means against Bacterial Preformed Biofilm

**Figure 7:**
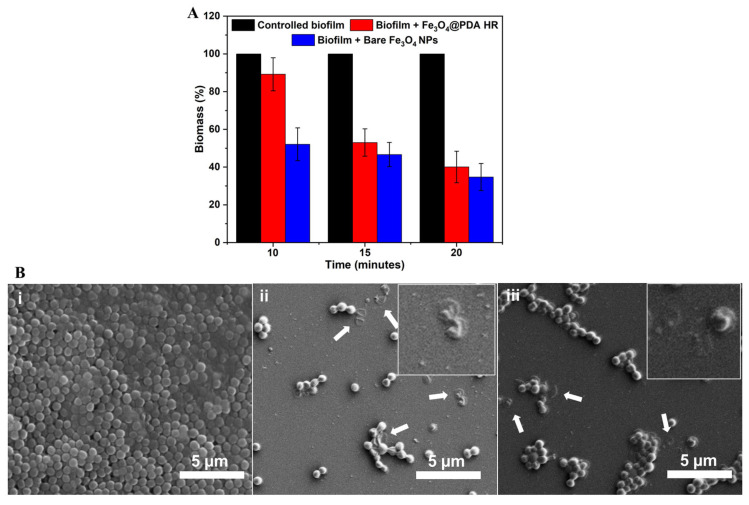
Reduction of pre-formed biofilm biomass upon exposure to rotational magnetic field (**A**), and SEM images of pre-formed biofilm upon exposure to rotational magnetic field (**B**): biofilm only (**i**), biofilm with added Fe_3_O_4_ NPs (**ii**) and biofilm with added Fe_3_O_4_@PDA HR (**iii**).

Preliminary experiments were carried out to prove whether Fe_3_O_4_ NPs could disrupt preformed bacterial biofilm under a rotational magnetic field. A preformed *S. aureus* biofilm was prepared and incubated with bare Fe_3_O_4_ NPs. After removal of un-attached Fe_3_O_4_ NPs, the treated biofilm was exposed to a rotational field at the speed of 2000 rpm. Strikingly, the biomass of preformed biofilm significantly declined to 52.0 ± 8.7%, 46.6 ± 6.4%, and 34.7 ± 7.2% after 10-, 15- and 20-min exposure to the magnetic rotational field, respectively (Figure 7A).

The disruption of biofilm caused by the movement of Fe_3_O_4_ NPs under the magnetic field was also examined using SEM imaging. As seen in Figure 7(Bii), the biofilm treated with bare Fe_3_O_4_ NPs was significantly reduced under the rotational magnetic field. The breakage of the cell membrane and the leakage of the internal cell content caused by Fe_3_O_4_ NPs was observed (as indicated with white arrows). This shows that Fe_3_O_4_ NPs induce damage to pathogenic bacteria either from within the interior in terms of biofilm’s uptake of Fe_3_O_4_ NPs or the exterior in case they escape from dead cells and disrupt adjacent cells under an applied magnetic field.

The use of Fe_3_O_4_@PDA HR to induce damage to *S. aureus* biofilm was then examined in the same way. After 10 min of exposure to the rotational magnetic field, the *S. aureus* biomass biofilm declined slightly by approximately 10%. However, the biomass was reduced dramatically to 53.0 ± 7.2% and 40.0 ± 8.4% after prolonged exposure times of 15 and 20 min, respectively (Figure 7A). This slow start phenomenon could suggest that the engulfment of the PDA wall might hinder the movement of Fe_3_O_4_ NP; hence, in order to break and escape from PDA HR carriers, longer exposure times to the rotational magnetic field might be required. The results indicate that Fe_3_O_4_@PDA HR has a similar effect to bare Fe_3_O_4_ NPs on disrupting biofilms in terms of inducing damage to cell walls, which could also be observed under SEM as indicated by white arrows in Figure 7(Biii). 

**Figure 8:**
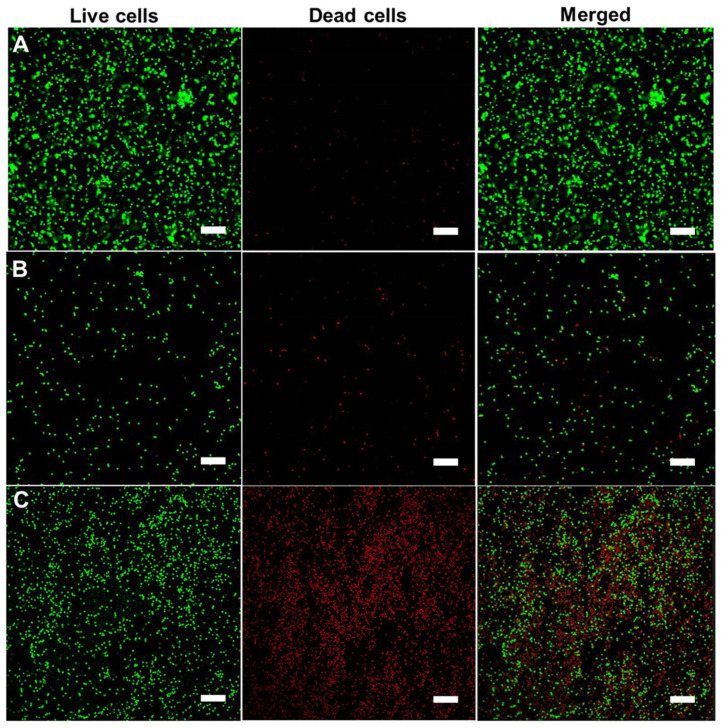
Live/dead cell staining of *S. aureus* biofilms under different treatment: control preformed biofilm (**A**), preformed biofilm treated with bare Fe_3_O_4_ NPs (**B**) and preformed biofilm treated with Fe_3_O_4_ @PDA HR (**C**) under rotational magnetic field (Scale bar = 10 µm).

To further confirm the above observation, the live/dead cell staining method was employed. As seen in Figure 8, in the case of bare Fe_3_O_4_, the fluorescent signals from biofilms post treatment are lower than those of the control experiment as well as the biofilm treated with Fe_3_O_4_@PDA HR. This might imply that the rotational force of the bare Fe_3_O_4_ under magnetic field might cause the detachment of the biofilm from the utilized substrate along with its wall disruption property to eliminate preformed biofilm. Furthermore, a significant amount of cell death was observed in either the biofilm treated with bare Fe_3_O_4_ or Fe_3_O_4_@PDA HR after exposure to a magnetic field, which further confirms the feasibility of utilizing magnetite at the nanoscale to control the growth of bacteria as well as their biofilm.

### 2.5. Fe_3_O_4_@PDA HR against Pre-Formed Biofilm under NIR Laser Irradiation

So far, PDA has been well-known for its excellent photothermal conversion, which has been utilized in various studies [26,27,28]. Taking this into consideration, further experiments exploiting the photothermal conversion effect of Fe_3_O_4_@PDA HR against preformed *S. aureus* biofilm was conducted.

**Figure 9:**
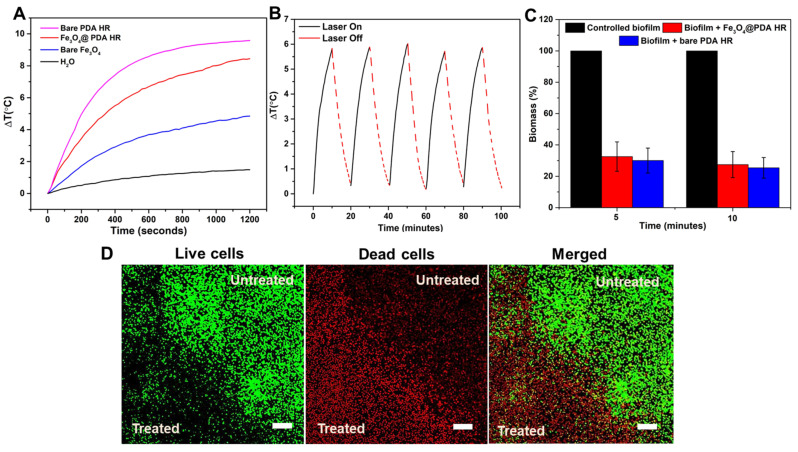
Photothermal conversion performance of bare Fe_3_O_4_ and Fe_3_O_4_@PDA HR (**A**) and photostability of Fe_3_O_4_@PDA HR (**B**); Reduction of preformed biofilm biomass upon laser irradiation (**C**) and live/dead cell staining of biofilm treated with Fe_3_O_4_@PDA HR upon exposure to NIR laser (Scale bar = 10 µm) (**D**).

To examine the photothermal conversion efficacy, a similar amount of materials was dispersed in water, and exposed to NIR laser irradiation at the wavelength of 795 nm. The water temperature containing either bare Fe_3_O_4_ or Fe_3_O_4_@PDA HR escalates upon the exposure to NIR laser irradiation at the wavelength of 795 nm. Specifically, after 20 min of laser irradiation, the temperature of water containing Fe_3_O_4_@PDA HR rises significantly by 8.4 °C, whereas the figure of bare Fe_3_O_4_ NPs was just around 4.9 °C (Figure 9A). The difference in temperature increase indicated that the presence of PDA enhances the heat conversion capacity of Fe_3_O_4_ NPs. However, the heat conversion of Fe_3_O_4_@PDA HR seems to be less effective compared to the bare hollow PDA HR (8.4 vs. 9.6 °C). This could be attributed to the presence of Fe_3_O_4_ NPs within the PDA network, which might reduce the synergistically acoustic vibration between PDA walls. In addition, the photostability of Fe_3_O_4_@PDA HR was quite stable even after being exposed to the laser source multiple times (Figure 9B).

As presented in Figure 9C, the biomass of preformed biofilms after the uptake of either bare PDA HR or Fe_3_O_4_@PDA HR particles was significantly diminished upon NIR laser treatment. In detail, the biomass of the biofilm treated with bare PDA HR and Fe_3_O_4_@PDA HR reduced to nearly 30.0 ± 8% and 32.6 ± 9%, respectively, from an initial 100% biomass of controlled biofilm after 5 min of NIR laser irradiation, and a further reduction in biomass to around 25.0 ± 6.6% and 27.5 ± 8.3%, respectively, could be observed within the next 5 min of laser treatment. Even though bare PDA HR has a better photothermal conversion compared to that of Fe_3_O_4_@PDA HR, these two materials have no significant performance difference for the elimination of preformed *S. aureus* biofilms. The elimination of preformed *S. aureus* biofilms using Fe_3_O_4_@PDA HR via NIR laser irradiation was further assessed by CLSM. A significant contrast between the untreated area and the area that received NIR treatment within biofilm could be observed (Figure 9D). The heat conversion capability of Fe_3_O_4_@PDA HR was triggered via laser treatment, and induced hyperthermia to bacterial cells, resulting in cell death as indicated by red signals, which represent the staining of nuclei of dead cells by propidium iodide [29,30], whereas virtually no effect on the growth of bacterial cells in the untreated area was observed, as indicated by strong green fluorescent signals, that had arisen from the metabolism of the SYTO 9 dye by the living cells [31].

Compared to previous studies on the use of Fe_3_O_4_ for biofilm eradication [18,32], the utilization of PDA HR to encapsulate and deliver monodisperse Fe_3_O_4_ NPs has several advantages as it could simultaneously co-deliver therapeutic drugs in a controllable manner. In addition, PDA based material could facilitate the localization of Fe_3_O_4_ NPs and optimize their quantity being uptaken by a particular cell of interest through the binding of catechol and amine groups distributed on PDA surface to the dopamine receptor family, D2DR as previously suggested by Liu’s group [33]. Even though the use of Fe_3_O_4_ filled PDA HR for biofilm elimination in this study was just carried out on gram positive bacteria as a proof of concept, the obtained results were quite promising. Hence, it is note-worthy for further study to focus on its potential application for different biofilms formed by different pathogenic bacteria or even cancerous cell lines in vitro and in vivo.

## 3. Materials and Methods

### 3.1. Materials and Reagents

Dopamine hydrochloride (DA), ammonia hydroxide (NH_4_OH, 28–30%), ethanol (EtOH), 2-propanol (iPrOH), tetraethyl orthosilicate (TEOS), cetyltrimethylammonium bromide (CTAB), Pluronic F127, 3-(aminopropyl) triethoxysilane (APTES), iron (III) chloride hexahydrate (FeCl_3_·6H_2_O), ethyl acetate and succinic acid were purchased from Sigma-Aldrich, Sydney, Australia.

### 3.2. Synthesis of Mesoporous Silica Rods (MSR) and Its Carboxylic Functionalization

The fabrication of MSR followed a procedure which was previously employed by Yang et al. [34]. Briefly, an amount of 0.615 g Pluronic F127 and 0.8925 g of CTAB were dissolved in a mixture of 80 mL H_2_O and 75 mL NH_4_OH (2.5 wt%) under stirring. Upon dissolution, 3 mL of TEOS were added to the as-prepared mixture. The reaction mixture was vigorously mixed for 2 min, followed by aging for 3 h under static conditions. The MSR was collected and washed several times prior to calcination at 550 °C for 2 h.

Grafting of the carboxylic acid group onto the surface of the silica was performed following a procedure, which was presented by An et al. [35] with some modifications. In brief, 200 mg of bare MSR was refluxed in 10 mL of toluene and 1 mL of APTES under stirring at 70 °C for 18 h to obtain amine functionalized MSR. After that, the amine functionalized MSR was dissolved in 20 mL of dimethylformamide (DMF) with the aid of sonication, followed by the addition of 20 mL 0.1 M succinic acid in DMF. The reaction was refluxed at 70 °C overnight under stirring. The carboxylic MSR particles were then collected via centrifugation and washed with DMF and ethanol, respectively.

#### Synthesis of Amine Functionalized Monodisperse Fe_3_O_4_ NPs

The synthesis of monodisperse Fe_3_O_4_ NPs was achieved through thermal decomposition of an iron precursor, iron (III) oleate. At first, the iron (III) oleate complex was synthesized according to a published procedure described by Park et al. [36] with some modifications. In detail, 9.133 g of sodium oleate (30 mmol) and 2.7 g of FeCl_3_·H_2_O (10 mmol) were dissolved completely in a solution containing 13.5 mL H_2_O, 20 mL EtOH and 32.8 mL hexane. The solution was then heated to 70 °C and blanketed with an argon atmosphere for 3 h. Upon the completion, the obtained waxy iron oleate that formed was washed three times with distilled water in a separatory funnel. The remaining hexane was volatilized naturally in the dish, and the final iron oleate complex was dried at 60 °C under vacuum.

After that, the synthesis of monodisperse Fe_3_O_4_ NPs followed a procedure which was previously described by Zhao et al. [37] with some minor modifications. In brief, 1.6 g iron oleate, 220 µL oleic acid, and 120 µL distilled H_2_O were mixed with 20 mL of 1-octadecene. The resulting solution was degassed and heated up to 320 °C for 2 h under inert atmosphere. After a predetermined time of 30 min, the solution was cooled down to room temperature and a volume of 60 mL *i*-PrOH was added to precipitate the nanoparticles. The nanoparticles were separated by centrifugation and washed 3 times with ethanol.

To further enhance the dispersibility of the obtained Fe_3_O_4_, 150 mg of Fe_3_O_4_ NPs were dispersed in 300 mL of EtOH containing 2 mL of H_2_O, which was followed by the addition of 100 µL of APTES. The reaction mixture was left overnight under magnetic stirring. The amine grafted Fe_3_O_4_ nanoparticles were magnetically separated and washed several times with EtOH and H_2_O.

### 3.3. Grafting Amine Functionalized Fe_3_O_4_ onto Carboxylic Functionalized MSR

Typically, 100 mg of carboxylic acid functionalized MSR and 200 mg of amine functionalized Fe_3_O_4_ were mixed in 40 mL of ethyl acetate. The coupling reaction was left at room temperature for 3 h under shaking. The collected Fe_3_O_4_ grafted MSR particles were collected via magnetic separation and washed several times with ethyl acetate prior drying at 60 °C under vacuum.

### 3.4. Fabrication of Fe_3_O_4_ NPs Filled PDA HR

An amount of 100 mg Fe_3_O_4_ grafted MSR was dispersed in a mixture comprising 190 mg of dopamine, 130 mL *i*-PrOH and 7 mL NH_4_OH. The coating process was left for 24 h under shaking at RT. The PDA coated silica particles were obtained by magnetic separation and washed several times with EtOH. The obtained PDA coated Fe_3_O_4_@MSR was then dispersed in H_2_O for 48 h to remove the silica cores. At the final step, Fe_3_O_4_@PDA HR was collected using magnetic separation and washed several times with H_2_O.

### 3.5. Loading Fosfomycin to Fe_3_O_4_@PDA HR

Typically, 100 mg of as-prepared Fe_3_O_4_@PDA HR was dispersed in 20 mL of fosfomycin solution (1 mg/mL) in sterilized H_2_O and left stirring overnight. After that, the drug loaded PDA HR were magnetically separated and washed several times with sterile H_2_O to remove unbound fosfomycin molecules. A liquid chromatography setup fitted with a mass spectrometer (LC-MS) was used to quantify the loading of fosfomycin. The experimental detection of fosfomycin via LC-MS was conducted using the direct injection mode with a mobile phase containing 20 mM ammonium acetate and methanol at a volumetric ratio of 50:50. The flow rate was fixed at 5 µL/min for all samples.

### 3.6. Stimuli Triggered Release of Fosfomycin 

#### 3.6.1. pH Mediated Drug Release Profile

An aliquot of 1 mL of fosfomycin loaded Fe_3_O_4_@PDA HR (10 mg/mL) was dispersed into 20 mL of 20 mM ammonium acetate at pH 7 and pH 5. After a predetermined time of every 30 min, 1 mL of release media was collected for LC-MS quantification, and 1 mL of fresh ammonium acetate was added to the release media.

#### 3.6.2. NIR Laser Irradiation Mediated Drug Release Profile

In a typical experiment, an aliquot of 3 mL of 20 mM ammonium acetate at pH 7 containing fosfomycin loaded Fe_3_O_4_@PDA HR (1 mg/mL) was employed. After 15-min intervals the cuvette was irradiated with a NIR laser light at a wavelength of 795 nm with a power density of 0.13 W/cm^2^ for 15 min. Before and after laser treatment, 1 mL of release media was collected and subjected to the LC-MS measurement. The release of fosfomycin from Fe_3_O_4_@PDA HR under the same conditions without laser treatment was used as a control.

#### 3.6.3. Release of Fosfomycin under Exposure to Rotational Magnetic Field

An aliquot of 1.0 mL of fosfomycin loaded Fe_3_O_4_@PDA HR (10 mg/mL) was dispersed into 20 mL of 20 mM ammonium acetate at pH 7 and exposed to a rotational magnetic field at a rotational speed of 2000 rpm. After a predetermined time, 1 mL of release media was collected for LC-MS measurement along with the addition of 1 mL of fresh buffer to the release media. The release of fosfomycin from Fe_3_O_4_@PDA HR under the same conditions without exposure to the magnetic field was used as a control.

### 3.7. Determination of Minimal Inhibitory Concentration of Free Fosfomycin and Fosfomycin Loaded Fe_3_O_4_@PDA HR against S. aureus 

An overnight cultured suspension of *Staphylococcus aureus* bacteria was diffused in fresh media of Mueller–Hinton broth (MHB) and the absorbance was adjusted to be within the range of 0.08–0.13 at a wavelength of 600 nm. Then, 100 µL of adjusted bacterial suspension was diluted in tubes containing 10 mL of fresh MHB to obtain bacterial suspensions of 10^6^ CFU/mL. A serial dilution of fosfomycin ranging from 512 µg/mL to 1 µg/mL was set-up in a 96-well microtiter plate (Corning, NY, USA) with the initial concentration being at 1 mg/mL, followed by the addition of 75 µL of diluted bacterial suspension into each well. For the growth control, 100 µL of diluted bacterial suspension was employed and wells containing only fresh medium broth were used as a negative control. In case of fosfomycin loaded Fe_3_O_4_@PDA HR, a serial dilution of a stock of fosfomycin loaded Fe_3_O_4_@PDA HR particles ranging from 5 mg/mL to 0.15 mg/mL was prepared by using the initial concentration of fosfomycin loaded Fe_3_O_4_@PDA HR of 10 mg/mL. After 24 h incubation, the absorbance of each well after incubation was measured at the wavelength of 595 nm using a microplate reader (POLARstar, Omega, BMG LABTECH, Ortenberg, Germany) to determine the MIC value of free fosfomycin and fosfomycin loaded Fe_3_O_4_@PDA HR against *S. aureus*.

#### Evaluating Anti-Biofilm Formation Activity of Fosfomycin Loaded Fe_3_O_4_@PDA HR via Metabolic Assay

An aliquot of 100 µL of fosfomycin loaded PDA suspension was pipetted to final concentrations of 0.25, 0.5 or 1 × MIC on a microtiter plate, followed by the addition of 100 µL of bacterial suspension at a concentration of 10^6^ CFU per mL, which was supplemented with glucose 1% beforehand. The microtiter plates were incubated at 37 °C for 24 h to allow the biofilm formation. After that, the culture medium was carefully removed, and the bacterial biofilm was left on the plate and dried out at 60 °C for 30 min. Subsequently, 100 µL of phosphate buffer saline (PBS) was added to each well, followed by the addition of 5 µL of prepared 3-(4,5-dimethylthiazol-2-yl)-2,5-diphenyltetrazolium bromide tetrazolium reduction assay (MTT) solution. Then, the plate was incubated at 37 °C for another 3 h before measuring ODs at the wavelength of 570 nm. The percentage of inhibition of peptide was calculated by:

$$\%\;inhibition=\frac{OD\;of\;growth\;control\;well-OD\;of\;well\;incubated\;with\;drug\;or\;drug\;loaded\;PDA}{OD\;of\;growth\;control\;well\;}\times 100$$

### 3.8. Evaluating the Effect of Fe_3_O_4_@ PDA HR against Pre-Formed Biofilm under Magnetic Rotation Field

In brief, *S. aureus* biofilm was firstly created by adding 100 µL of bacterial suspension at a concentration of 10^6^ CFU per mL supplemented with glucose 1% on microtiter plate with 24 h incubation at 37 °C. The culture medium was later carefully removed, and bacterial biofilm was left on the plate. At this stage, 100 µL of Fe_3_O_4_@ PDA HR suspension (1 mg/mL) was incubated with the preformed biofilm for 1 h for the biofilm uptake of nanoparticles. After that, the supernatant was removed, and the biofilms were washed with PBS to remove the excess nanoparticles. As-prepared samples were primarily exposed to a rotating neodymium magnet for 10–20 min, and the rotational speed of the magnetic field was maintained at 2000 rpm prior to the addition of 100 µL of fresh PBS and MTT reagent. A preformed biofilm of *S. aureus* treated with Fe_3_O_4_@ PDA HR suspension without undergoing further treatment was employed as a control. The biomass of remaining preformed biofilm after treatment was calculated by:

$$\%\;remained\;biomass=\frac{\;OD\;of\;treated\;biofilm\;}{OD\;of\;control\;biofilm}\times 100$$

#### Evaluating the Effect of Fe_3_O_4_@ PDA HR against Pre-Formed Biofilm upon Laser Irradiation

The detailed procedure was as above, but instead of exposing to rotational magnetic field, a laser source at the wavelength of 795 nm with the power intensity at 0.5 W/cm^2^ was applied to the treated biofilm for 5 or 10 min prior the addition of 100 µL of fresh PBS and MTT reagent.

### 3.9. Live/Dead Cell Staining for Antibiofilm Effect

Biofilms of *S. aureus* were first grown on a glass-bottom petri dish (FluoroDisk). The viability of biofilms after being exposed to different stimuli was then evaluated via the LIVE/DEAD™ BacLight™ Bacterial Viability Kit (Invitrogen^TM^, ThermoFisher, Melbourne, Australia). Specifically, 200 μL of stain solution containing 3 μL of SYTO^®^ 9 stain and 3 μL of propidium iodide stain predispersed in 1 mL of sterilized water was added to the treated biofilms, followed by a 30-min incubation in the dark. Excess staining on the biofilm was then removed and rinsed with water. Then, the live/dead stained biofilm was observed under confocal laser scanning microscopy (Olympus Fluoview 1000 IX81, Olympus Corporation, Melbourne, Australia).

### 3.10. Material Characterization

Scanning Electron Microscope (SEM) images and energy dispersive X-ray (EDX) spectroscopy were obtained on a field emission scanning electron microscope (FeSEM, ZEISS SUPRA 40VP, Carl Zeiss NTS GmbH, Oberkochen, Germany) at an acceleration voltage of 3 kV. Transmission electron microscopy (TEM) was performed using a JEM 2100F (JEOL Ltd., Akishima, Japan) at room temperature at an accelerating voltage of 200 kV. The crystal information of the samples was collected via an X-ray diffractometer (Bruker, D8-Advance, Karlsruhe, Germany) with Cu Ka radiation. The quantification of fosfomycin was carried out using the Agilent 1290 Infinity II UHPLC apparatus coupled with a 6545 Q-TOF MS (Agilent Technologies, Santa Clara, CA, USA). The optical characterizations were performed using a UV-Vis spectrophotometer (Halo RB-10, Dynamica Pty Ltd., Victoria, Australia). Confocal Laser Scanning Microscopy (CLSM) images were performed using Olympus Fluoview 1000 IX81 confocal laser scanning microscope, operated using 100× oil-immersion objective combined with 5× optical zoom.

## 4. Conclusions

The successful fabrication of Fe_3_O_4_ filled PDA HR was confirmed by various characterization techniques including SEM, EDX, XRD and TEM. The capability of using obtained Fe_3_O_4_@PDA HR for fosfomycin loading and controlled release under various stimulations was fully examined. Of note, the incorporation of Fe_3_O_4_ NPs within PDA HR not only offers magnetic guidance property for the drug carrier but also facilitates the release of the payload upon exposure to the rotational magnetic field. More importantly, for the first time we demonstrated that the encapsulation of monodisperse Fe_3_O_4_ NPs within PDA HR could serve as a mechanical means to disrupt the structure of preformed biofilms upon exposure to the rotational magnetic field. 

Undoubtedly, Fe_3_O_4_@PDA HR can be utilized efficiently either as a carrier platform for chemotherapeutic agents or as a mechanical means to disrupt the structure of preformed bacterial biofilms. This study offers an alternative way of making use of the drug carrier platform as a weaponized material rather than its conventional purpose for drug delivery to fight against bacterial biofilm in the era of antibiotic-resistant bacteria.

## Data Availability

Not applicable.
